# Supplementary material for: Identification of photoperiod-induced specific miRNAs in the adrenal glands of Sunite sheep (Ovis aries)
Source: Front Vet Sci. 2022 Jul 22;9:888207. doi: 10.3389/fvets.2022.888207 (PMC9354845; doi:10.3389/fvets.2022.888207)
Supplement: Supplementary file 1 [file Data_Sheet_1.ZIP › Supplementary file/miRDeep2 file.zip!/miRDeep2 file/Know_Structure/LP42A1/oar-mir-323a.pdf]

miRBase precursor : oar-mir-323a
Total read count : 1072
oar-miR-323a-5p read count : 4
oar-miR-323a-3p read count : 1067
remaining reads : 1

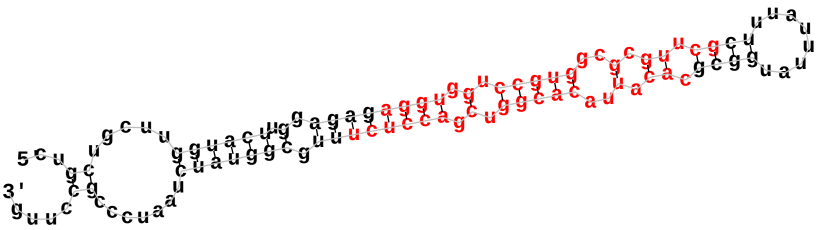

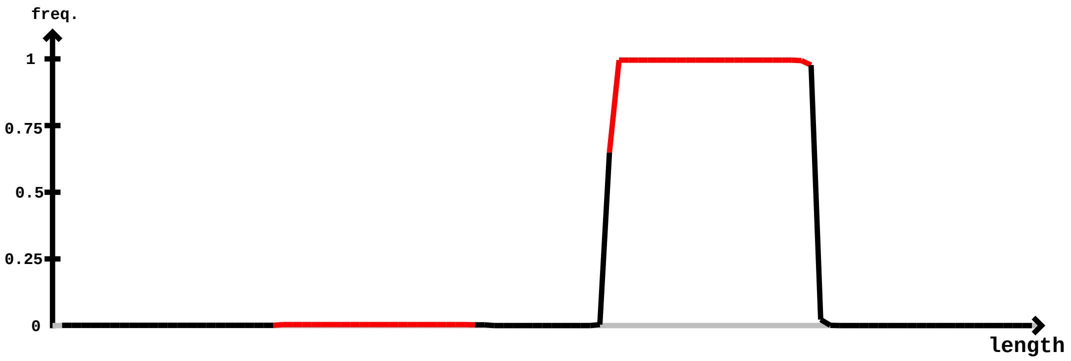


oar-miR-323a-5p

oar-miR-323a-3p

```
5'- cugcugcuugguacuuggagagaggugguccguggcgcguucgcuuuauuuauggcgcacauuacacggucgaccucuuugcgguaucuaaucccgccuug   -3'    exp
    .........((((((.(.((((((((.(.(((((..(.((.((((........)))).)).)..))))).).)))))))).))))))).............    reads     mm        sample
    cugcugcuugguacuuggagag...............................................................................    1         0         seq
    ......................aggugguccguggcgcguuc...........................................................    1         0         seq
    ......................aggugguccguggcgcguucgc.........................................................    3         0         seq
    .......................................................cgcacauuacacggucgaccuc........................    1         0         seq
    .......................................................cgcacauuacacggucgaccucu.......................    3         0         seq
    ........................................................gcacauuacacggucgaccu.........................    2         0         seq
    ........................................................gcacauuacacggucgaccuc........................    12        0         seq
    ........................................................gcacauuacacggucgaccucu.......................    665       0         seq
    ........................................................gcacauuacacggucgaccucuu......................    13        0         seq
    .........................................................cacauuacacggucgaccuc........................    5         0         seq
    .........................................................cacauuacacggucgaccucu.......................    355       0         seq
    .........................................................cacauuacacggucgaccucuu......................    10        0         seq
    .........................................................cacauuacacggucgaccucuuu.....................    1         0         seq
```
